# Fournier Gangrene Caused by *Candida albicans* in an Infant After Cardiac Surgery

**DOI:** 10.1007/s11046-016-0086-4

**Published:** 2016-11-02

**Authors:** Radoslaw Jaworski, Ninela Irga-Jaworska, Łukasz Naumiuk, Maciej Chojnicki, Ireneusz Haponiuk

**Affiliations:** 1 Department of Pediatric Cardiac Surgery, Copernicus Hospital in Gdansk, Al. Jana Pawla II 50, 80-462 Gdansk, Poland; 2 Department of Pediatrics, Hematology and Oncology, Medical University of Gdansk, ul. Debinki 7, 80-210 Gdansk, Poland; 3 Department of Clinical Microbiology, Hospital of the Medical University of Gdansk, ul. Debinki 7, 80-952 Gdansk, Poland; 4 Chair of Physiotherapy, Department of Rehabilitation and Kinesiology, Gdansk University of Physical Education and Sport, ul. Kazimierza Górskiego 1, 80-336 Gdansk, Poland

**Keywords:** Fournier gangrene, *Candida albicans* infection, Pediatric cardiac surgery, Congenital heart defects, Echinocandins, Fluconazole

## Abstract

Fournier gangrene is a rare, rapidly progressive, life-threatening condition. We report a 23-day-old boy with pulmonary atresia and ventricular septal defect treated surgically, who developed Fournier gangrene. Emergency surgery was performed with tissue sampling for microbiological examination. *Candida albicans* was confirmed; caspofungin followed by fluconazole was administered with excellent results.

## Introduction

Fournier gangrene (FG) is a rare, rapidly progressive, life-threatening condition with necrotizing fasciitis of the perineum first mentioned by Baurienne in 1764, however, described and named after Jean Alfred Fournier and his report from the year 1883 [1, 2]. The disease mostly affects patients between 50 and 60 years of age and is uncommon in children. The typical flora involves polymicrobial bacteria, most commonly *Escherichia coli*, *Proteus*, *Staphylococcus*, *Enterococcus* and anaerobes; however, single cases of fungal etiology in adults were reported [2–5]. The risk factors of FG developing include diabetes, immunosuppression, chronic kidney disease, local trauma, urethral stricture and genitourinary infections [3]. It is essential to diagnose FG as early as possible, because the infection can rapidly spread from local necrosis to adjacent organs, leading to sepsis and death; mortality rates of 7.5–88% are reported [5–7]. We report a case of a male infant, who developed fungal FG after cardiac surgery.

## Case Report

**Fig. 1:**
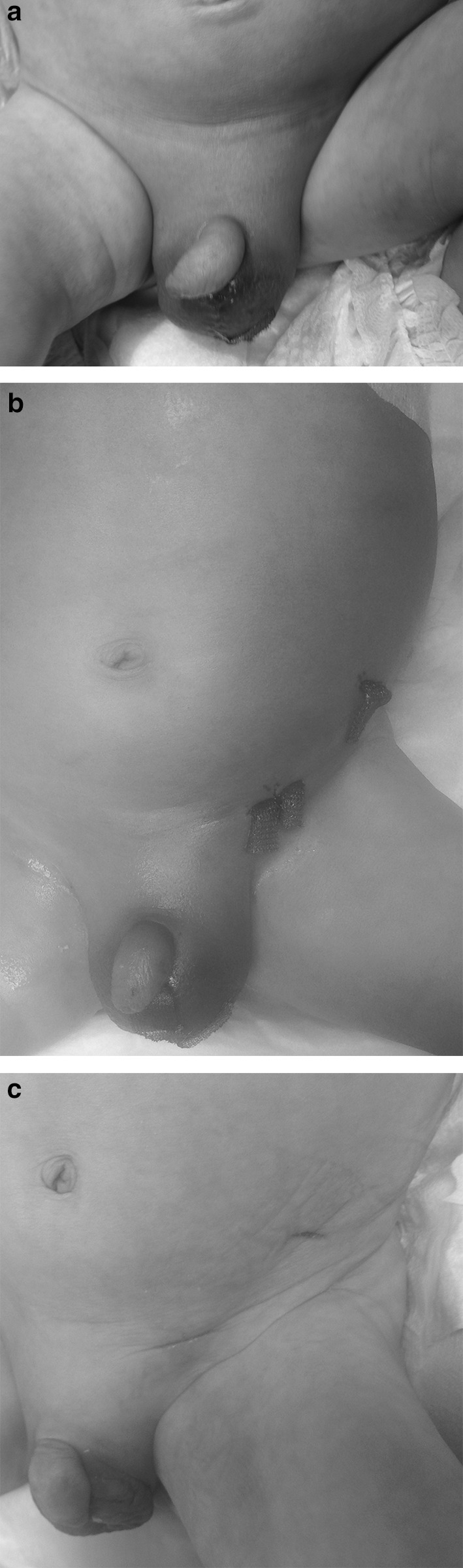
Fournier gangrene due to *Candida albicans* in an infant after cardiosurgical treatment erythema and edema of scrotum, groins with extension toward left axilla after scrotum surgical examination and debridement (**a**), status post-second surgical intervention after next 24 h (**b**) and significant local improvement after 14 days of antifungal therapy (**c**)

A 23-day-old male new-born weighting 3.1 kg with pulmonary atresia and ventricular septal defect was admitted to the Department of Pediatric Cardiac Surgery for treatment. The child was born hypotrophic (2.7 kg) at 38 weeks of gestation, and his mother suffered from myasthenia gravis. Routinely performed microbiological screening at admission revealed that the child’s throat was colonized with methicillin-resistant *Staphylococcus aureus* (MRSA) and rectum with *Candida albicans*. Before the admission for surgery, there was difficulty with urinary catheterization probably due to urethral. However, after few attempts before the operation, a smaller sized catheter was successfully placed. Perioperative antibiotic prophylaxis consisted of vancomycin and gentamycin. After diagnostic catheterization and detailed pulmonary vessels, anatomy description unifocalization with aorto-pulmonary shunts was performed bilaterally (Gore-tex^®^, 3.5 mm in diameter). Significant blood steal syndrome occurred postoperatively, resulting in insufficient organ perfusion and requiring prolonged intensive care unit stay (ICU). Other complications included: prolonged mechanical ventilation, digestive problems requiring total parenteral nutrition, cholestasis and diaper rash. Reoperation due to left-sided shunt problems was necessary on the 7th postoperative day. After 2 weeks, on the 15th day after the first surgery clinical examination revealed painful erythema and edema of the scrotum; however, the child was not febrile. Scrotal examination showed mucopurulent tissue infiltration, and local debridement was performed; samples for microbiological examination were taken. Complete blood count examination revealed 11.16 G/l of white blood count, whereas C-reactive protein (CRP) and procalcitonin (PCT) were 68 mg/l and 0.55 ng/ml, respectively. *Candida* score was estimated as three points (colonization, total parenteral nutrition and surgery). Antibacterial (meropenem, vancomycin) and antifungal (fluconazole) empirical therapy was initiated. Within the next 24 h erythema and edema were spreading over both groins and toward the left axilla (Fig. 1). FG was diagnosed clinically, and the next emergency surgery was performed relieving edema in order to prevent microthrombosis of small subcutaneous vessels and progressive necrotizing fasciitis. Tissue sampling for further microbiological examination was also performed. *C. albicans* was confirmed in all microbiological cultures from tissue samples, and blood samples, however, were negative for *Candida*. Because of instable clinical status, the child was transferred to ICU. Caspofungin was started (loading dose of 70 mg/m^2^, then 50 mg/m^2^) and continued for 14 days followed by fluconazole with excellent results (Fig. 1). Follow-up period at 6 months remains uneventful.

## Discussion

FG is a rare condition with the incidence of 1.6 cases per 100,000 males adult per year [10]. The disease mostly affects patients between 50 and 60 years of age, and in children FG is not common. However, FG is a fulminant and life-threatening disease, because although proper treatment mortality in these groups of patients remains high, mortality rates of 7.5–88% are reported [2, 5–8]. A literature review found only <60 pediatric cases of FG worldwide, with 66% of those in infants younger than 3 months [9].

Commonly, the etiology of FG is polymicrobial and the infection is most often caused by three or more microorganisms: *E. coli*, *Proteus*, *Staphylococcus*, *Enterococcus* and anaerobes [3–5]. However, single-case reports describing FG caused by fungi are reported. *C. albicans*, *Candida glabrata* and *Rhisopus microsporus* etiology are described in adults [5, 10, 11]. Our case report is unique because to the best of our knowledge there is no report of solitary *C. albicans* FG in children.

It is essential to diagnose FG as early as possible, because the infection can rapidly spread from local necrosis to adhering organs, causing sepsis and death. The diagnosis of FG is based on clinical symptoms such as intense pain, pruritus and tenderness in the genitalia, usually associated with edema of the overlying skin. It is worth emphasizing the role of round the clock access to microbiological laboratory with wide spectrum of diagnostics examination. The guidelines for FG treatment recommend hemodynamic stabilization, aggressive wide surgical debridement and empirical broad-spectrum antibiotics therapy. Also hyperbaric oxygen therapy is recognized as an additional form of FG treatment [2]. However, worldwide increase in fungal infections incidence results in suggestion of some authors to add the empirical antifungal treatment, especially in patients at risk for fungal infections. This concerns mainly patients with primary and secondary immunodeficiencies. Fluconazole therapy could be a reasonable therapeutic option; however, due to wide-spread use of azoles a large number of non-albicans *Candida* species isolates in recent years suggest that echinocandins may be a good choice for empiric therapy, especially in hemodynamic unstable patients [3, 5, 12]. Also combined therapy of different antifungal therapeutics groups may be successful if monotherapy fails; echinocandin and liposomal amphotericin are suggested in this setting [13].

In our case, secondary immunodeficiency in hypotrophic infant was probably caused by significant blood steal syndrome that occurred postoperatively, resulting in vital organs hypoperfusion. Another risk factor for FG was preoperative traumatic urinary catheterization. However, the first symptoms of FG showed relatively late, on 15th postoperative day. Our patient was colonized with methycillin-resistant *S. aureus* (MRSA) as well as with *C. albicans* preoperatively; nevertheless, twice as surprisingly the fungi were an etiological factor for FG. One could explain this phenomenon that the perioperative antibiotic prophylaxis appeared effective against MRSA, while no prophylaxis against fungi was used. In our case, rapid diagnosis based on clinical signs, broad-spectrum empirical antibiotics and aggressive surgical approach with debridement and tissue sampling for microbiological identification of an etiological agent which guided our pharmacotherapy led to final therapeutic success.

In conclusion, the successful treatment of FG depends on early diagnosis and aggressive etiological management. Therapeutic strategy includes hemodynamic stabilization, broad-spectrum antibiotics and wide surgical debridement with maximum excision of all necrotic tissues. General increase in fungal infections and the incidence of fungal colonization worldwide may suggest that empiric antifungal treatment should also be considered in FG.
